# Exploring Pump–Probe Response in Exciton–Biexciton Quantum Dot–Metal Nanospheroid Hybrids

**DOI:** 10.3390/mi16121319

**Published:** 2025-11-25

**Authors:** Spyridon G. Kosionis, Dimitrios P. Alevizos, Emmanuel Paspalakis

**Affiliations:** Materials Science Department, School of Natural Sciences, University of Patras, 265 04 Patras, Greece; phy5833@ac.upatras.gr

**Keywords:** exciton–biexciton, pump–probe response, metal nanospheroid, plasmonic nanostructure, semiconductor quantum dot

## Abstract

We study the optical susceptibility of a CdSe-based semiconductor quantum dot with a cascade exciton–biexciton configuration, which is coupled via the Coulomb interaction to a gold spheroidal nanoparticle, in the presence of a nearly resonant strong pump field and a weak probe field. We take both fields’ polarization vectors to be parallel to the interparticle axis, derive the equations of motion for the density matrix, and proceed with a perturbative expansion approach to calculate the components of the density matrix associated with the effective optical susceptibility, which describes processes to first order in the probe field and to all orders in the pump field. We present spectra of the effective susceptibility and examine their dependence on the metal nanoparticle’s geometric characteristics for various interparticle distances and pump field detunings, under both one- and two-photon resonance conditions. The role of the biexciton energy shift is also studied. Lastly, we introduce a dressed-state picture to elucidate the origin of the observed spectral features. Our calculations reveal that reducing the interparticle distance and increasing the metal nanoparticle aspect ratio enhance the exciton–plasmon coupling, leading to pronounced resonance splitting, spectral shifts, and broadened gain regions. Prolate nanoparticles aligned with the field polarization exhibit the strongest coupling and the widest gain bandwidth, whereas oblate geometries produce nearly overlapping resonances. Under exact resonance, the probe displays zero absorption with a negative dispersion slope, indicating slow-light behavior. These results demonstrate the tunability of hybrid CdSe-Au nanostructures for designing nanoscale optimal amplifiers, modulators, and sensors.

## 1. Introduction

Over the past few years, research on light–matter interactions at the nanoscale has recognized hybrid nanosystems composed of metal nanoparticles (MNPs) and semiconductor quantum dots (SQDs) as a promising platform for the engineering of nonlinear optical properties. The unique behavior of these coupled exciton–plasmon systems has attracted growing interest in a broad range of disciplines, including quantum optics and nanophotonics. One of the primary reasons for their popularity is the tunability of their optical response by structural and electromagnetic parameters, which allows control over energy transfer, information processing, and light manipulation at subwavelength scales with high precision. A key domain of exploration in these hybrids centers around their nonlinear optical behavior. Some of the phenomena that have been studied are modified Rabi oscillations [1,2], nonlinear Fano resonances in energy transfer and exciton-induced transparency [3,4,5,6], plasmonic-induced transparency [7,8,9], modification of plasmonic nanolasers and energy transfer due to nonlocal effects [10,11], nonlinear energy transfer in multi-level quantum SQDs near plasmonic nanostructures [12,13,14,15], as well as strongly modified exciton transfer [1,16,17,18], high-order harmonic generation [19], altered optical bistability [20,21,22], and resonance fluorescence in SQDs near MNPs [23,24]. Other effects include plasmonic meta-resonances in WS_2_–metallic nanoantenna systems [25], second- and third-order nonlinearities [26,27,28], and quantum interference effects [29,30] in SQDs coupled to spherical MNPs. Moreover, optical effects have also been studied in SQDs coupled to core-shell plasmonic nanostructures [31,32,33] and to metallic nanospheroids [34,35,36].

Special attention has been given in the interaction of SQD-MNP systems with pump–probe fields [37,38,39,40,41,42,43,44,45]. In most of these studies, the MNP is treated as a homogeneous nanosphere, while the SQD is typically assumed to support only a single excitonic transition. Specifically, Lu and Zhu were the first to show that coupling an SQD to a spherical MNP could alter the cross-Kerr nonlinearity [37], while also enabling slow light propagation [38]. These breakthroughs triggered a wave of research into a variety of nonlinear effects in similar nanoscale setups. To mention a few examples, Sadeghi reported lasing without inversion, highlighting the impact of plasmon-assisted meta-resonances in shaping absorption and gain spectra [39,40], while Li et al. addressed optical bistability in cross-Kerr nonlinearity [41] and four-wave parametric amplification [42]. An additional layer of understanding was provided by the work of Kosionis and Paspalakis and others through a series of studies focusing on the control of pump–probe response [43], four-wave mixing (FWM) [44,45], and self-Kerr nonlinearities [46].

Though most of these studies focused on the ground-exciton transition, subsequent studies broadened the scope of investigation to the exciton–biexciton transition via two-photon absorption in SQD–MNP nanoassemblies interacting with a single electromagnetic field [12,47,48,49]. In these works, the MNP was typically modeled as a nanosphere or a more complex core–shell structure. Recent studies have demonstrated that exciton-to-biexciton transitions, under the influence of a strong pump and a weak probe field, can induce additional coherence effects and nonlinearities in such hybrid systems [50]. Building on this foundation, a more recent study [51] investigated the pump–probe optical response and FWM in quantum dot exciton–biexciton–MNP hybrids, where both ground–exciton and exciton–biexciton transitions are simultaneously excited, while other studies have also analyzed strong coupling effects in multi-level SQDs coupled to MNPs [52,53]. In addition, novel optical properties, due to exciton–plasmon interplay on the surface of ellipsoidal MNPs have also been explored, including enhanced energy transfer rates [8], the generation of plasmonic metaresonances [25], the manipulation of the polarization of plasmonic fields [34,35,36], and the control of optical bistability [54,55].

To the best of our knowledge, the coherent pump–probe response of a strongly driven exciton–biexciton SQD system coupled to a non-spherical MNP has not yet been explored. Addressing this gap is the focus of the present work, exploring how these dual transition pathways influence the spectral features under various excitation and coupling conditions. In particular, we consider the nonlinear optical response of a hybrid nanosystem comprising a spheroidal MNP and an SQD modeled as a three-level cascade exciton–biexciton quantum system. In this work, we develop a theoretical framework for describing the optical response of a semiconductor quantum dot coupled to an ellipsoidal metal nanoparticle under the action of strong and weak optical fields. This approach extends previous studies on spherical geometries by introducing shape anisotropy and orientation-dependent coupling, which enable direct control over the local-field enhancement, effective Rabi frequency, and exciton–plasmon interaction strength. The model demonstrates how variations in the MNP aspect ratio, interparticle distance, and field detuning lead to tunable spectral shifts, gain enhancement, and dispersion modification. The results provide new physical insight into plasmon-induced nonlinearities and establish a geometry- and field-controlled approach for designing nanoscale optical switches, amplifiers, and sensors based on SQD-MNP hybrid structures.

The remainder of the paper is organized as follows. In Section 2, we provide the fundamental theoretical framework. More specifically, we employ the density-matrix formalism to calculate the effective optical susceptibility of the SQD and implement the dressed-state picture to predict the positions of the resonances. In Section 3, we analyze the numerical results concerning the dispersion and absorption/gain spectra of the SQD for different MNP geometries and values of the center-to-center distance. Finally, Section 4 provides a summary of the main findings.

## 2. Methods

**Figure 1:**
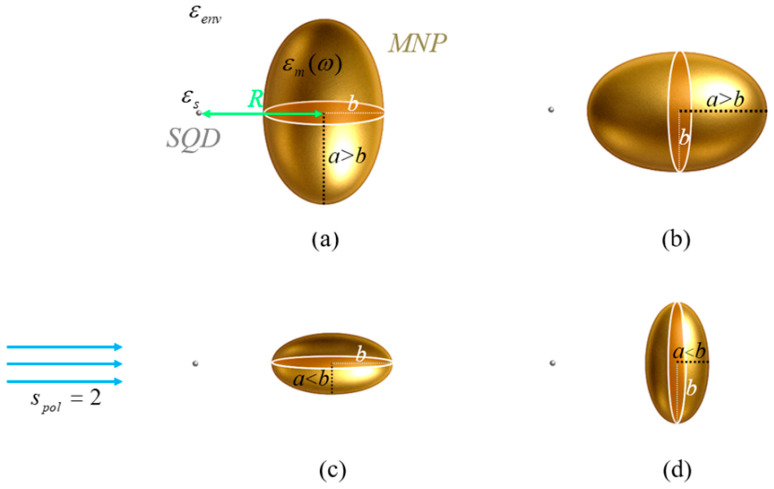
The hybrid nanosystem comprises an SQD coupled with a spheroidal MNP with fixed equatorial semiaxis length b and polar semiaxis of variable length a interacting with a field polarized along the symmetry axis of the nanostructure. Specifically, configurations (**a**,**b**) illustrate prolate nanospheroids (q=a/b>1), while (**c**,**d**) depict oblate nanospheroids (q<1). The relative dielectric permittivity for the SQD and the environment are denoted by $\varepsilon_{S}$ and $\varepsilon_{env}$, respectively, the dielectric function $\varepsilon_{m}(\omega )$ describes the gold MNP and the center-to-center distance is symbolized with *R*.

The hybrid nanosystem examined consists of an SQD with relative dielectric permittivity $\varepsilon_{S}$ and a gold spheroidal MNP with dielectric function $\varepsilon_{m}(\omega )$, both placed in a medium with relative permittivity $\varepsilon_{env}$, at a center-to-center distance R, as illustrated in Figure 1. The length of the polar semiaxis of the spheroid is denoted as a, while that of the radial semiaxes as b. The value of the aspect ratio q=a/b classifies the spheroid into three cases: prolate spheroid for q>1, oblate spheroid for q<1, and sphere for q=1.

The MNP is treated as a classical particle, while the energy level structure of the SQD, which is treated quantum mechanically, corresponds to an exciton–biexciton cascade energy-level scheme, with ground state |1〉 single-exciton state |2〉 and biexciton state |3〉. The hybrid nanostructure interacts with two linearly polarized fields: $\vec{E_{a}}(t)=\hat{\varepsilon }E_{a}\cos(\omega_{a}t)$ and $\vec{E_{b}}(t)=\hat{\varepsilon }E_{b}\cos(\omega_{b}t)$, the first being a strong pump field and the second a weak probe field. The polarization vector, same in both fields, lies parallel to the interparticle axis.

The applied electric fields $\vec{E_{a}}(t)$ and $\vec{E_{b}}(t)$ drive the interband transitions |1〉→|2〉→|3〉 between the ground, exciton and biexciton states of the SQD, respectively. These fields induce an oscillating dipole moment in the SQD, which emits an electromagnetic field that polarizes the nearby MNP and excites its surface plasmons. The plasmonic oscillations generate a strong, broadband near field that feeds back onto the SQD through the Coulomb (dipole–dipole) interaction. As a result, the SQD excitation is modified and a coherent coupling is established between the exciton and biexciton transitions and the plasmonic mode of the MNP. This bidirectional interaction leads to the formation of a hybrid exciton and must be incorporated into the SQD Hamiltonian, which, under the dipole approximation, takes the form:

(1) $$H=\sum_{n=1}^{3}E_{n}|n\rangle \langle n|-\mu \sum_{j=a,b}E_{jSQD}\left(|1\rangle \langle 2|+|2\rangle \langle 3|+c.c.\right).$$

**Figure 2:**
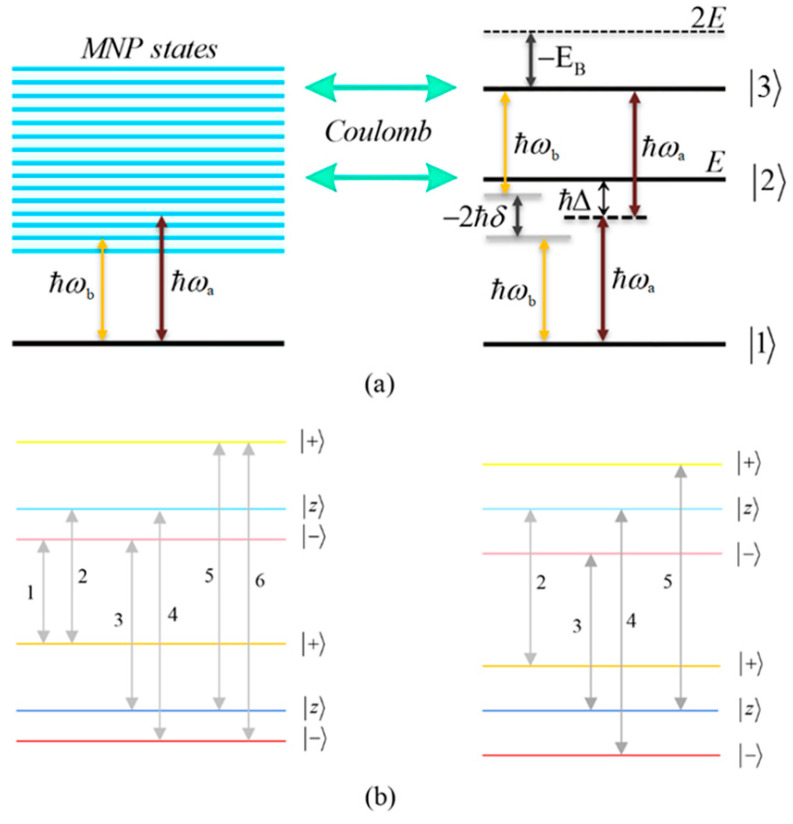
(**a**) Illustration of the cascade energy level diagram representing the exciton–biexciton system. The ground state is labeled as |1〉. The single-exciton state and the biexciton state are represented by |2〉 and |3〉, with corresponding energies E and $2E+E_{B}$, respectively, where $E_{B}$ is the biexciton binding energy. Δ is the frequency detuning of the pump field from the |1〉↔|2〉 resonance and $\delta =\omega_{b}-\omega_{a}$ indicates the frequency offset between the pump and probe fields, whose frequencies are $\omega_{a}$ and $\omega_{b}$, respectively. The surface plasmons excited on the MNP provide a continuous spectral response. (**b**) This dressed-state framework illustrates, under the two-photon resonance condition $\left(2\hbar \Delta +E_{B}=0\right)$, the possible transition pathways within the exciton–biexciton system. The left diagram describes the off-resonant case, where the pump field is positively detuned (Δ>0), while right diagram depicts the resonant condition with zero detuning (Δ=0).

The first term describes the SQD in the absence of the MNP and the incident electromagnetic fields, with energy levels $E_{1}=0$, $E_{2}=\hbar \omega_{0}$ and $E_{3}=2\hbar \omega_{0}+E_{B}$, with $E_{B}$ symbolizing the biexciton energy shift and $\omega_{0}$ indicating the single-exciton resonance frequency, as shown in panel (a) of Figure 2. The second term takes into account the interaction of the SQD with the pump and the probe fields in the presence of the MNP, with $E_{jSQD}$ being the pump (j=a) or probe (j=b) field inside the SQD:

(2) $$E_{jSQD}=\hbar /\mu \left[\Omega_{j}e^{-i\omega_{j}t}+G_{j}\left({\tilde{\rho }}_{21}(t)+{\tilde{\rho }}_{32}(t)\right)\right]+c.c.\;.$$

Here, μ denotes the electric dipole transition matrix element, which is assumed to be the same for the |1〉↔|2〉 and |2〉↔|3〉 transitions, while ${\tilde{\rho }}_{21}(t)$ and ${\tilde{\rho }}_{32}(t)$ are the density matrix elements associated with these transitions. The terms $\Omega_{j}$ represent the Rabi frequencies, which are composed of two terms:

(3) $$\Omega_{j}=\Omega_{0j}\left(1+\frac{s_{pol}\gamma_{j}ab^{2}}{R^{3}}\right).$$

The former term $\Omega_{0a}=\mu E_{j}/\left(2\hbar \varepsilon_{effS}\right)$ corresponds to the direct coupling of the SQD to the applied fields, while the latter, to the field produced by the MNP due to its interaction with the fields. The self-interaction factors $G_{j}$ are associated with the Förster energy transfer between the MNP and the SQD, and are given by the following formula:

(4) $$G_{j}=\frac{1}{4\pi \varepsilon_{env}}\frac{s_{pol}^{2}\gamma_{j}ab^{2}\mu^{2}}{\hbar \varepsilon_{effS}^{2}R^{6}}.$$

Since the applied fields are parallel to the interparticle axis, we set $s_{pol}=2$. Moreover, we have $\gamma_{j}=\left[\varepsilon_{m}(\omega_{j})-\varepsilon_{env}\right]/\left\{3\xi_{pol/eq}\left[\varepsilon_{m}(\omega_{j})-\varepsilon_{env}\right]+3\varepsilon_{env}\right\}$ and $\varepsilon_{effS}=\left(2\varepsilon_{env}+\varepsilon_{S}\right)/3\varepsilon_{env}$, with $\xi_{pol/eq}$ being the depolarization factor [34,35]. When the applied electric fields are polarized along the polar axis of the spheroid, the depolarization factor is expressed as

(5a) $$\xi_{pol}=\frac{1}{1-q^{2}}\left\{1-q\frac{\arcsin\left[1-q^{2}\right]}{\sqrt{1-q^{2}}}\right\}.$$

If the applied fields are polarized along the radial axis, the depolarization factor is expressed as

(5b) $$\xi_{eq}=\left(1-\xi_{pol}\right)/2.$$

The above equations show that: for prolate spheroids $0<\xi_{pol}<1/3$ and $1/3<\xi_{eq}<1/2$, for oblate spheroids $1/3<\xi_{pol}<1$ and $0<\xi_{eq}<1/3$, while, for the limiting case of a sphere, we take $\xi_{pol}=\xi_{eq}=1/3$.

Next, we define the slowly varying density matrix elements $\rho_{21}={\tilde{\rho }}_{21}e^{i\omega_{a}t}$, $\rho_{32}={\tilde{\rho }}_{32}e^{i\omega_{a}t}$, $\rho_{31}={\tilde{\rho }}_{31}e^{2i\omega_{a}t}$ and introduce the Hamiltonian into the Liouville–von Neumann–Lindblad equation. After considering that $\rho_{nm}=\rho_{mn}^{*}$, $\rho_{nn}={\tilde{\rho }}_{nn}$ and applying the rotating wave approximation (RWA), we derive a set of six differential equations that describe the dynamics of the system. Furthermore, using population conservation, i.e., $\sum_{n=1}^{3}\rho_{nn}=1$, the total number of linearly independent differential equations is reduced to five, given by

(6a) $$\begin{array}{l} {\dot{\rho }}_{12}(t)=i\Delta \rho_{12}(t)-i\left\{\Omega_{a}^{*}+G_{a}^{*}\left[\rho_{12}(t)+\rho_{23}(t)\right]\right\}\left[\rho_{11}(t)-\rho_{22}(t)\right]\\ \;\;\;\;\;\;\;\;\;\;\;\;\;-i\left\{\Omega_{b}^{*}e^{i\delta t}+G_{b}^{*}\left[\rho_{12}(t)+\rho_{23}(t)\right]\right\}\left[\rho_{11}(t)-\rho_{22}(t)\right]\\ \;\;\;\;\;\;\;\;\;\;\;\;\;-i\left\{\Omega_{a}+G_{a}\left[\rho_{21}(t)+\rho_{32}(t)\right]\right\}\rho_{13}(t)\\ \;\;\;\;\;\;\;\;\;\;\;\;\;-i\left\{\Omega_{b}e^{-i\delta t}+G_{b}\left[\rho_{21}(t)+\rho_{32}(t)\right]\right\}\rho_{13}(t)-\gamma_{12}\rho_{12}(t), \end{array}$$

(6b) $$\begin{array}{l} {\dot{\rho }}_{23}(t)=i\left(\Delta +E_{B}/\hbar \right)\rho_{23}(t)\\ \;\;\;\;\;\;\;\;\;\;\;\;\;-i\left\{\Omega_{a}^{*}+G_{a}^{*}\left[\rho_{12}(t)+\rho_{23}(t)\right]\right\}\left[2\rho_{22}(t)+\rho_{11}(t)-1\right]\\ \;\;\;\;\;\;\;\;\;\;\;\;\;-i\left\{\Omega_{b}^{*}e^{i\delta t}+G_{b}^{*}\left[\rho_{12}(t)+\rho_{23}(t)\right]\right\}\left[2\rho_{22}(t)+\rho_{11}(t)-1\right]\\ \;\;\;\;\;\;\;\;\;\;\;\;\;+i\left\{\Omega_{a}+G_{a}\left[\rho_{21}(t)+\rho_{32}(t)\right]\right\}\rho_{13}(t)\\ \;\;\;\;\;\;\;\;\;\;\;\;\;+i\left\{\Omega_{b}e^{-i\delta t}+G_{b}\left[\rho_{21}(t)+\rho_{32}(t)\right]\right\}\rho_{13}(t)-\gamma_{23}\rho_{23}(t), \end{array}$$

(6c) $$\begin{array}{l} {\dot{\rho }}_{13}(t)=i\left(2\Delta +E_{B}/\hbar \right)\rho_{13}(t)+i\left\{\Omega_{a}^{*}+G_{a}^{*}\left[\rho_{12}(t)+\rho_{23}(t)\right]\right\}\left[\rho_{23}(t)-\rho_{12}(t)\right]\\ \;\;\;\;\;\;\;\;\;\;\;\;\;+i\left\{\Omega_{b}^{*}e^{i\delta t}+G_{b}^{*}\left[\rho_{12}(t)+\rho_{23}(t)\right]\right\}\left[\rho_{23}(t)-\rho_{12}(t)\right]-\gamma_{13}\rho_{13}(t), \end{array}$$

(6d) $$\begin{array}{l} {\dot{\rho }}_{11}(t)=i\left\{\Omega_{a}^{*}+G_{a}^{*}\left[\rho_{12}(t)+\rho_{23}(t)\right]\right\}\rho_{21}(t)\\ \;\;\;\;\;\;\;\;\;\;\;\;\;+i\left\{\Omega_{b}^{*}e^{i\delta t}+G_{b}^{*}\left[\rho_{12}(t)+\rho_{23}(t)\right]\right\}\rho_{21}(t)\\ \;\;\;\;\;\;\;\;\;\;\;\;\;-i\left\{\Omega_{a}+G_{a}\left[\rho_{21}(t)+\rho_{32}(t)\right]\right\}\rho_{12}(t)\\ \;\;\;\;\;\;\;\;\;\;\;\;\;-i\left\{\Omega_{b}e^{-i\delta t}+G_{b}\left[\rho_{21}(t)+\rho_{32}(t)\right]\right\}\rho_{12}(t)+\Gamma_{21}\rho_{22}(t), \end{array}$$

(6e) $$\begin{array}{l} {\dot{\rho }}_{22}(t)=i\left\{\Omega_{a}^{*}+G_{a}^{*}\left[\rho_{12}(t)+\rho_{23}(t)\right]\right\}\left[\rho_{32}(t)-\rho_{21}(t)\right]\\ \;\;\;\;\;\;\;\;\;\;\;\;\;+i\left\{\Omega_{b}^{*}e^{i\delta t}+G_{b}^{*}\left[\rho_{12}(t)+\rho_{23}(t)\right]\right\}\left[\rho_{32}(t)-\rho_{21}(t)\right]\\ \;\;\;\;\;\;\;\;\;\;\;\;\;-i\left\{\Omega_{a}+G_{a}\left[\rho_{21}(t)+\rho_{32}(t)\right]\right\}\left[\rho_{23}(t)-\rho_{12}(t)\right]\\ \;\;\;\;\;\;\;\;\;\;\;\;\;-i\left\{\Omega_{b}e^{-i\delta t}+G_{b}\left[\rho_{21}(t)+\rho_{32}(t)\right]\right\}\left[\rho_{23}(t)-\rho_{12}(t)\right]\\ \;\;\;\;\;\;\;\;\;\;\;\;\;+\Gamma_{32}\left[1-\rho_{11}(t)-\rho_{22}(t)\right]-\Gamma_{21}\rho_{22}(t). \end{array}$$

In the above equations, the parameter $\Delta =\omega_{0}-\omega_{a}$ is the detuning of the pump field from the |1〉↔|2〉 transition and $\delta =\omega_{b}-\omega_{a}$ is the frequency mismatch between the two applied fields, as illustrated in panel (a) of Figure 2. The quantities $\gamma_{nm}$ and $\Gamma_{nm}$ correspond to the dephasing and decay rate coefficients, respectively.

We proceed with our investigation, based on the assumption that the probe field is weak compared to the pump field $\left(\left|\Omega_{b}\right|<<\left|\Omega_{a}\right|\right)$. This implies that, while all orders of interaction of the pump field with the SQD need to be considered, in the case of the probe field, considering only the first order of its interaction with the SQD is a satisfactory approximation. Therefore, according to the first order approximation, we have $\rho_{nm}=\rho_{nm}^{\left(1\right)}+\Omega_{b}^{*}e^{i\delta t}\rho_{nm}^{\left(2\right)}+\Omega_{b}e^{-i\delta t}\rho_{nm}^{\left(3\right)}$, where $\left|\rho_{nm}^{\left(1\right)}\right|>>\left|\Omega_{b}^{*}\rho_{nm}^{\left(2\right)}\right|$, $\;\left|\Omega_{b}\rho_{nm}^{\left(3\right)}\right|$. The terms $\rho_{nm}^{\left(1\right)}$ describe the system in the absence of the probe field, while, the relations ${\left(\rho_{nm}^{\left(1\right)}\right)}^{*}=\rho_{mn}^{\left(1\right)}$ and ${\left(\rho_{nm}^{\left(2\right)}\right)}^{*}=\rho_{mn}^{\left(3\right)}$ are satisfied. The calculation of the $\rho_{nm}^{\left(k\right)}$ elements, with k=1,  2,  3, is achieved by solving numerically, in the steady-state limit, the differential equations derived by substituting the above series expansions in Equation (6a–e) and by properly equating the powers of $\Omega_{b}$ to the harmonics of δ. Especially important are the elements $\rho_{12}^{(3)*}$ and $\rho_{23}^{(3)*}$, calculated in the steady-state, since, the susceptibility for the SQD of the probe field in first-order, in the presence of the pump field, is given by the following expression:

(7) $$\chi_{SQD}^{\left(1\right)}=\frac{\Gamma \mu^{2}}{V\varepsilon_{0}\hbar \Omega_{b}}{\left(\rho_{12\;\;SS}^{\left(3\right)}+\rho_{23\;\;SS}^{\left(3\right)}\right)}^{*}.$$

Here, V, Γ and $\varepsilon_{0}$ denote the volume of the SQD, the optical confinement factor, and the dielectric constant of the vacuum, respectively.

Defining, now, the effective Rabi frequency as $\Omega_{eff}=\Omega_{a}+G_{tot}{\left[\rho_{12\;\;SS}^{\left(1\right)}+\rho_{23\;\;SS}^{\left(1\right)}\right]}^{*}$, with $G_{tot}=G_{a}+G_{b}$, helps us better understand the dynamics of the system, as its introduction into Equation (6a,b), in the steady-state, makes them appear phenomenologically linear, and resembling those of a single SQD in the presence of a pump–probe field [50]. Theoretical prediction of resonance positions in the spectra arising from nonlinear optical processes can be achieved using the dressed-state analysis method. The dressed states are eigenstates of the time-independent part of the Hamiltonian associated with the pump field. For a strong pump field, the population amplitudes satisfy the relation

(8) $$i\hbar \frac{d}{dt}\left[\begin{array}{c} a_{1}(t)\\ a_{2}(t)\\ a_{3}(t) \end{array}\right]=H_{a}(t)\left[\begin{array}{c} a_{1}(t)\\ a_{2}(t)\\ a_{3}(t) \end{array}\right],$$

where we set

(9) $$H_{a}(t)=\left[\begin{array}{ccc} 0 & Y & 0\\ Y & \hbar \omega_{0} & Y\\ 0 & Y & 2\hbar \omega_{0}+E_{B} \end{array}\right],$$

with $Y=-\hbar \left(\Omega_{eff}e^{-i\omega_{a}t}+\Omega_{eff}^{*}e^{i\omega_{a}t}\right)$.

If we apply the transformation relations $a_{1}(t)=b_{1}(t)$, $a_{2}(t)=b_{2}(t)e^{-i\omega_{0}t}$ and $a_{3}(t)=b_{3}(t)e^{-i(2\omega_{0}+E_{B}/\hbar )t}$, the newly defined population amplitudes $b_{n}(t)$ satisfy the differential equations

(10) $$i\hbar \frac{d}{dt}\left[\begin{array}{c} b_{1}(t)\\ b_{2}(t)\\ b_{3}(t) \end{array}\right]=H_{b}(t)\left[\begin{array}{c} b_{1}(t)\\ b_{2}(t)\\ b_{3}(t) \end{array}\right],$$

where under the RWA, we have

(11) $$H_{b}(t)=\left[\begin{array}{ccc} 0 & -\hbar \Omega_{eff}^{*}e^{-i\Delta t} & 0\\ -\hbar \Omega_{eff}e^{i\Delta t} & 0 & -\hbar \Omega_{eff}^{*}e^{-i(\Delta +E_{B}/\hbar )t}\\ 0 & -\hbar \Omega_{eff}e^{i(\Delta +E_{B}/\hbar )t} & 0 \end{array}\right].$$

Finally, the transformation relations $b_{1}(t)=c_{1}(t)$, $b_{2}(t)=c_{2}(t)e^{i\Delta t}$ and $b_{3}(t)=c_{3}(t)e^{i(2\Delta +E_{B}/\hbar )t}$ result in the time-independent Hamiltonian

(12) $$H_{c}=\left[\begin{array}{ccc} 0 & -\hbar \Omega_{eff}^{*} & 0\\ -\hbar \Omega_{eff} & \hbar \Delta & -\hbar \Omega_{eff}^{*}\\ 0 & -\hbar \Omega_{eff} & 2\hbar \Delta +E_{B} \end{array}\right].$$

Under the two-photon resonance condition $\left(2\hbar \Delta +E_{B}=0\right)$, the above Hamiltonian gives the following eigenvalues:

(13) $$\lambda_{z}=0\mathrm{and}\;\lambda_{\pm }=\hbar \left(\Delta \pm \sqrt{\Delta^{2}+8{\left|\Omega_{eff}\right|}^{2}}\right)/2,$$

which correspond respectively to the dressed state

(14) $$|z\rangle =\frac{|1\rangle -\left(\Omega_{eff}/\Omega_{eff}^{*}\right)|3\rangle }{\sqrt{2}},$$

a dark state, and to the dressed states

(15) $$|\pm \rangle =\frac{Z|1\rangle +4\left|\Omega_{eff}\right||2\rangle +Z^{*}|3\rangle }{2\sqrt{8{\left|\Omega_{eff}\right|}^{2}+\Delta^{2}\mp \Delta \sqrt{\Delta^{2}+8{\left|\Omega_{eff}\right|}^{2}}}},$$

with $Z=\left(\frac{\left|\Omega_{eff}\right|}{\Omega_{eff}}\right)\left(\Delta \mp \sqrt{\Delta^{2}+8{\left|\Omega_{eff}\right|}^{2}}\right)$. The bare states are related to the dressed states as follows

(16) |1〉|2〉|3〉=Ωeffλ−Ωeffκ−12Ωeffλ+Ωeffκ+2ℏΩeffκ−02ℏΩeffκ+Ωeff*λ−Ωeffκ−−Ωeff*2ΩeffΩeff*λ+Ωeffκ+|+〉|z〉|−〉,

where $\kappa_{\pm }=\hbar \sqrt{8{\left|\Omega_{eff}\right|}^{2}+\Delta^{2}\pm \Delta \sqrt{\Delta^{2}+8{\left|\Omega_{eff}\right|}^{2}}}$.

We can now express the Hamiltonian describing the coupling of the exciton–biexciton system with the probe field, in the dressed-state picture, as follows:

(17) $$\begin{array}{l} H^{\prime }=-\hbar \left[\Omega_{b}e^{-i\omega_{b}t}+G_{b}\left({\tilde{\rho }}_{21}+{\tilde{\rho }}_{32}\right)\right]\left\{4\hbar \Omega_{eff}\frac{\lambda_{-}}{\kappa_{-}^{2}}|+\rangle \langle +|+4\hbar \Omega_{eff}\frac{\lambda_{+}}{\kappa_{+}^{2}}|-\rangle \langle -|\right.\\ \;\;+2\hbar \Omega_{eff}\frac{\left(\lambda_{+}+\lambda_{-}\right)}{\kappa_{+}\kappa_{-}}|+\rangle \langle -|+2\hbar \Omega_{eff}\frac{\left(\lambda_{+}+\lambda_{-}\right)}{\kappa_{+}\kappa_{-}}|-\rangle \langle +|+\frac{2\hbar \left|\Omega_{eff}\right|}{\sqrt{2}\kappa_{-}}|z\rangle \langle +|\\ \;\;+\frac{2\hbar \left|\Omega_{eff}\right|}{\sqrt{2}\kappa_{+}}|z\rangle \langle -|\left.-\frac{2\hbar \Omega_{eff}^{2}}{\sqrt{2}\left|\Omega_{eff}\right|\kappa_{-}}|+\rangle \langle z|-\frac{2\hbar \Omega_{eff}^{2}}{\sqrt{2}\left|\Omega_{eff}\right|\kappa_{+}}|-\rangle \langle z|\right\}+c.c. \end{array}.$$

From the above Hamiltonian, for a positive field detuning (Δ>0), we can deduce that two transitions correspond to the case with δ=0. These transitions are described by the first two terms containing the operators |+〉〈+| and |−〉〈−|. This is consistent with the information presented in the left panel of Figure 2b depicting the possible transition pathways within the exciton–biexciton system. The next six terms correspond to the transitions for which a non-zero mismatch between the pump and the probe field frequencies is present. In the order they appear in the above Hamiltonian, these transitions occur for $\hbar \delta =\lambda_{+}+\left|\lambda_{-}\right|$, $-\lambda_{+}-\left|\lambda_{-}\right|$, $-\lambda_{+}$, $\left|\lambda_{-}\right|$, $\;\lambda_{+}$, $-\left|\lambda_{-}\right|$. Provided that both the one-photon and two-photon resonance conditions are satisfied, the Hamiltonian becomes

(18) $$\begin{array}{l} H\prime \prime =-\frac{\hbar }{2}\left[\Omega_{b}e^{-i\omega_{b}t}+G_{b}\left({\tilde{\rho }}_{21}+{\tilde{\rho }}_{32}\right)\right]\left\{-\sqrt{2}\frac{\Omega_{eff}}{\left|\Omega_{eff}\right|}|+\rangle \langle +|+\sqrt{2}\frac{\Omega_{eff}}{\left|\Omega_{eff}\right|}|-\rangle \langle -|\right.\\ \;\;\;\;\;\;\;\;+|z\rangle \langle +|+|z\rangle \langle -|\left.-\frac{\Omega_{eff}^{2}}{{\left|\Omega_{eff}\right|}^{2}}|+\rangle \langle z|-\frac{\Omega_{eff}^{2}}{{\left|\Omega_{eff}\right|}^{2}}|-\rangle \langle z|\right\}+c.c.. \end{array}$$

Here, the positions of the resonances detected on the spectra correspond to δ=0, $\pm \sqrt{2}\left|\Omega_{eff}\right|$. This limiting case reduces the number of resonance doublets to two. The positions of these doublets coincide, and hence, we only observe a single doublet of resonances in addition to the central (dark-state) resonance.

## 3. Parameters and Results

In the hybrid nanosystem examined in the present work, a CdSe-based SQD is characterized by a dielectric constant $\varepsilon_{S}=6\varepsilon_{0}$ [51] and an exciton energy $\hbar \omega_{0}=2.5\;\mathrm{eV}$. The decay and dephasing rates are assigned as $\Gamma_{21}=\Gamma_{32}=1/800{\;\mathrm{ps}}^{-1}$ and $\gamma_{12}=\gamma_{13}=\gamma_{23}=0.2{\;\mathrm{ps}}^{-1}$, respectively. Additionally, the dipole moment μ, assumed to be the same for both |1〉↔|2〉 and |2〉↔|3〉 transitions, is set to 0.65 enm [51]. In the strong confinement regime, the ratio of the optical confinement to the volume of the SQD, Γ/V, is taken equal to $5\times 10^{23}\;m^{-3}$.

All calculations are performed assuming the case of a gold MNP, characterized by the dielectric function $\varepsilon_{m}(\omega )$, as given in [56], and the MNP-SQD system is embedded in a vacuum environment $\left(\varepsilon_{env}=\varepsilon_{0}\right)$. The length of the radial semiaxis b of the spheroidal MNP is kept constant at 7.5 nm, while that of the polar semiaxis is defined by the relation a=q⋅b, q symbolizing the aspect ratio. This entails that, since q is set equal to 3/2 and 1/4, for a prolate and oblate MNP respectively, the length of the polar semiaxis takes the values of 11.25 nm and 1.9 nm, as shown in the configurations of Figure 1.

**Figure 3:**
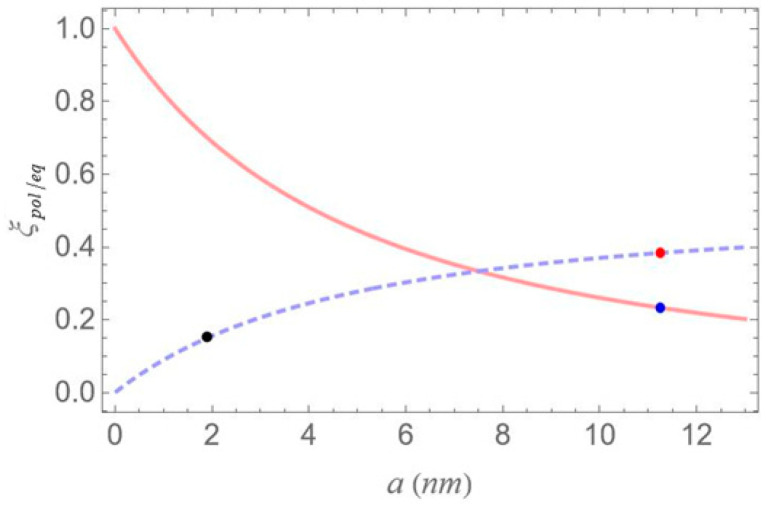
The depolarization factors $\xi_{pol}$ and $\xi_{eq}$, plotted as a function of the length of the polar semiaxis, a. The solid orange (/dashed blue) curve represents the depolarization factor when the MNP’s polar axis is aligned parallel (/perpendicular) to the polarization direction of the incident field, $\xi_{pol}$ and $\xi_{eq}$, as calculated by Equation (5a,b), respectively. For values of a<7.5 nm (where a<b), the MNP has the shape of an oblate spheroid, while for a>7.5 nm (where a>b), the MNP is a prolate spheroid. The red, blue and black markers correspond to the configurations (a), (b) and (c) of Figure 1, respectively.

In Figure 3, we present the depolarization factor, $\xi_{pol/eq}$, as a function of the polar semiaxis a. The dashed blue curve corresponds to the case where the applied electric fields are polarized along the radial axis of the MNP, whereas the solid orange curve corresponds to the case of the electric fields being polarized along its polar axis. The blue, red, and black markers on the curves correspond to the three distinct configurations we examine: the red marker represent configuration (a) from Figure 1, the blue corresponding to configuration (b), and the black to configuration (c).

**Figure 4:**
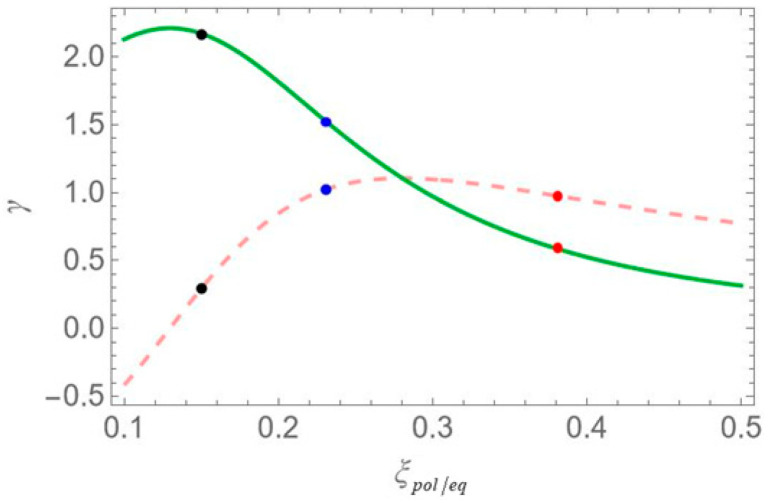
The parameters Re(γ) and Im(γ), depicted with the dashed orange curve and the solid green curve, respectively, are plotted as functions of the depolarization factors $\xi_{pol}$ and $\xi_{eq}$ depending on whether the MNP’s polar axis is aligned parallel or perpendicular to the polarization direction of the incident field. As in Figure 3, the red, blue and black markers correspond to configurations (a), (b) and (c) of Figure 1.

**Figure 5:**
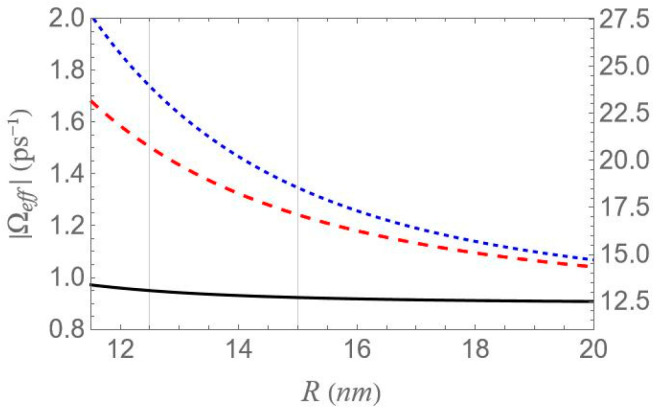
The absolute value of the effective Rabi frequency, $\left|\Omega_{eff}\right|$, as a function of the interparticle distance, R, for each one of the distinct configurations depicted in Figure 1, assuming pump field Rabi frequencies equal to $0.9{\;\mathrm{ps}}^{-1}$ (left axis) and $12.4{\;\mathrm{ps}}^{-1}$ (right axis). The dashed red, the dotted blue and the solid black curves refer to configurations (a), (b) and (c) of Figure 1.

In Figure 4, we plot the real and imaginary parts of the parameter γ, as functions of the depolarization factor, with the dashed orange curve corresponding to the real and the solid green to the imaginary part. In Figure 5, we display the absolute value of the effective Rabi frequency $\left|\Omega_{eff}\right|$ as a function of the interparticle distance R, with the dashed red curve portraying configuration (a), the dotted blue configuration (b) and the solid black configuration (c). Two vertical lines mark the interparticle distances of 12.5 and 15 nm, which coincide with two of the three distances considered in our study, the third being 100 nm. The scale on the left axis of the plot corresponds to a low free-space pump field Rabi $\left(\Omega_{0a}=0.9{\;\mathrm{ps}}^{-1}\right)$, while the right axis represents a higher value $\left(\Omega_{0a}=12.4{\;\mathrm{ps}}^{-1}\right)$. In all cases, the intensity of the probe field is assumed to be four orders of magnitude lower than that of the pump field. For all sets of the parameters investigated in the present study, the transitions associated with the resonances of the hybrid exciton lie in the ‘bright’ meta-resonance region.

## 4. Discussion

In this section, we analyze the spectra of both the real and imaginary components of the effective optical susceptibility of the SQD–MNP hybrid system under strong optical pumping conditions. All spectra presented in this section are plotted directly from Equations (7)–(10) and correspond to absolute (not-normalized) values of the real and the imaginary parts of the first-order optical susceptibility. The analysis is carried out for various interparticle distances, MNP eccentricity values, and MNP–SQD configurations. We consider both on-resonant and off-resonant probe fields, with detunings selected to match one-photon and two-photon resonant transitions.

**Figure 6:**
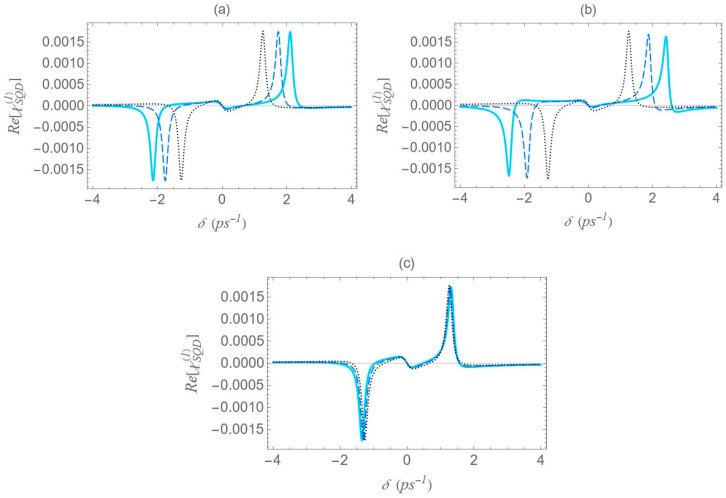
Dispersion spectrum of the SQD, Re[χSQD(1)], under the one-photon (Δ=0) and the two-photon $\left(2\Delta +E_{B}/\hbar =0\right)$ resonance conditions, for Rabi frequency $\Omega_{0a}=0.9{\;\mathrm{ps}}^{-1}$, decay rates $\Gamma_{21}=\Gamma_{32}=1/800{\;\mathrm{ps}}^{-1}$, $\Gamma_{31}=0$, and dephasing rates $\gamma_{12}=\gamma_{13}=\gamma_{23}=$$0.2{\;\mathrm{ps}}^{-1}$, for different center-to-center distances (solid cyan curve: R=12.5 nm, dashed blue curve: 15 nm and dotted black curve: 100 nm). The rest of the parameters used are a=7.5 nm, $\varepsilon_{S}=6\varepsilon_{0}$, $\varepsilon_{env}=\varepsilon_{0}$, $s_{a}=2$, μ=0.65 enm and $\hbar \omega_{0}=2.5\;\mathrm{eV}$. Panels (**a**), (**b**) and (**c**) correspond to the configurations (a), (b) and (c) of Figure 1, respectively.

In Figure 6, we present the dispersion spectra of the probe field, calculated by the real part of the optical susceptibility of the SQD, Re[χSQD(1)], as a function of the pump–probe frequency mismatch, δ, for specific configurations of Figure 1, under both the one-photon and two-photon resonance conditions $\left(\Delta =E_{B}=0\right)$, in the low pump field intensity regime $\left(\Omega_{0a}=0.9{\;\mathrm{ps}}^{-1}\right)$. Panels (a), (b) and (c) correspond to the configurations (a), (b) and (c) of Figure 1, respectively. The solid cyan curve corresponds to the interparticle distance R of 12.5 nm, the dashed blue to the distance of 15 nm, and the dotted black to that of 100 nm. As predicted by the dressed-state analysis (see the right diagram of Figure 2b), when the one-photon and two-photon resonance conditions are simultaneously satisfied, only three resonances remain in the spectrum: one central peak at zero pump–probe frequency mismatch, and two sidebands located at $\delta =\pm \sqrt{2}\left|\Omega_{eff}\right|$ (maximum and minimum, respectively).

**Figure 7:**
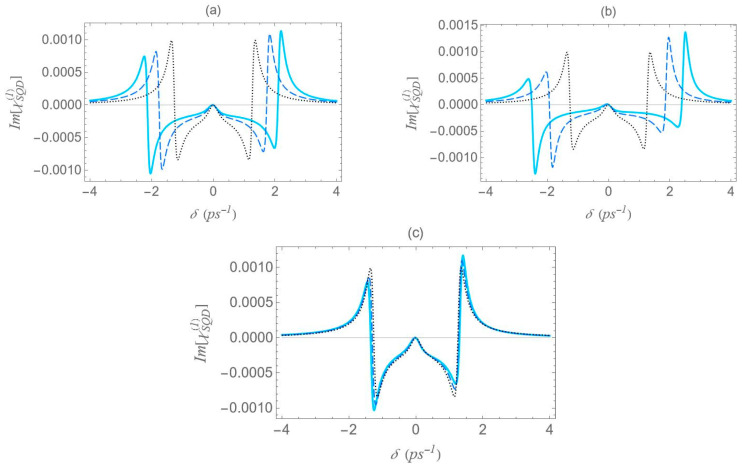
Absorption spectrum of the SQD, Im[χSQD(1)], under the one-photon (Δ=0) and the two-photon $\left(2\Delta +E_{B}/\hbar =0\right)$ resonance conditions, for Rabi frequency $\Omega_{0a}=0.9{\;\mathrm{ps}}^{-1}$. The rest parameters are the same as in Figure 6.

However, it should be noted that the accuracy of these analytical expressions holds primarily in the high-pump regime, where the pump field intensity significantly exceeds the decay and dephasing rates, which is indeed the case throughout our study. Otherwise, deviations are to be expected. At large interparticle distances, all spectra exhibit antisymmetric lineshape profiles; however, as the distance decreases this symmetry gradually breaks. As the interparticle distance decreases, the distance between the resonances $\left(2\sqrt{2}\left|\Omega_{eff}\right|\right)$ increases, since, as shown in Figure 5, the absolute value of the effective Rabi frequency $\left|\Omega_{eff}\right|$ decreases with the increase of R, for any fixed configuration. For configuration (c), the spectral curves nearly overlap, regardless of the interparticle distance. This observation is consistent with the solid black curve in Figure 5, which indicates a weak dependence of $\left|\Omega_{eff}\right|$ on the interparticle distance for this configuration. Regardless of interparticle distance and MNP geometry, all spectra exhibit a zero crossing at the center, accompanied by a negative slope. This feature indicates a substantial reduction in the group velocity of the probe field that is combined with zero absorption, as confirmed by Figure 7, where δ=0 corresponds to ImχSQD(1)=0. The negative slope of the dispersion at the transparency point signifies a reduced group velocity of the probe field, consistent with the dressed-state phase response of the strongly driven exciton–biexciton system.

Examining the different configurations, we deduce that at a fixed interparticle distance, the resonances are spread within a wider range of values along the δ axis for configuration (b) compared to (a), while configuration (c) exhibits the least broadening along the same axis. For the set of parameters used in our calculations, the variation in the $\left|\Omega_{eff}\right|$ parameter across the different MNP geometries is mainly attributed to the modification of the second term of $\Omega_{a}$, namely $\Omega_{0a}s_{pol}\gamma_{a}ab^{2}/R^{3}$. For configurations (c) and (d), with polar semiaxis length a=1.9 nm, the MNP volume $\left(\propto ab^{2}\right)$ is negligible compared to the one of configurations (a) and (b), with a=11.25 nm. Hence, the value of $\left|\Omega_{eff}\right|$, at low interparticle distances, can be regarded as insignificant, while it also shows a weak dependence on the R parameter. Between configurations (a) and (b) that share the same MNP volume, the absolute value of $\gamma_{a}$, as seen in Figure 4, is higher for configuration (b). This explains why the dotted blue curve lies above the dashed red curve in Figure 5 and accounts for the broader spectral profile of configuration (b). Furthermore, when the applied fields are polarized perpendicular to the symmetry axis of the hybrid nanostructure $\left(s_{pol}=-1\right)$, we found that at the smallest interparticle distance investigated (R=12.5 nm), the difference in $\left|\Omega_{eff}\right|$ between configurations (b) and (c) is 3.6 times lower than that observed in the case with $s_{pol}=2$. Hence, the spectra are not expected to exhibit a strong dependence on the MNP’s geometrical characteristics or interparticle distance. For this reason, we opted not to include those results in the present study. We now briefly discuss two additional cases for which the spectra are not presented in this study. In the case of an oblate nanospheroid with aspect ratio q=1/4, illustrated in configuration (d), the spectral lines are found to coincide perfectly across all interparticle distances. For a perfectly spherical MNP, the spectral lineshapes closely resemble those observed for the prolate nanospheroid of configuration (a), with aspect ratio q=3/2.

**Figure 8:**
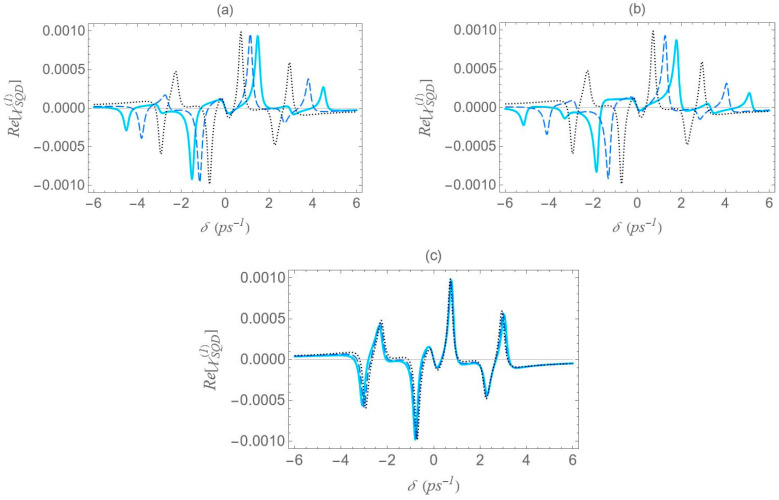
Dispersion spectrum of the SQD, Re[χSQD(1)], under the two-photon resonance condition $\left(2\Delta +E_{B}/\hbar =0\right)$, for Rabi frequency $\Omega_{0a}=0.9{\;\mathrm{ps}}^{-1}$, pump field detuning $\Delta =1.5{\;\mathrm{ps}}^{-1}$, and biexciton energy shift $E_{B}/\hbar =-3{\;\mathrm{ps}}^{-1}$.

In Figure 8, we present the dispersion spectra of the probe field, Re[χSQD(1)], for the same configurations of Figure 1 as above, under the two-photon resonance condition $\left(2\Delta +E_{B}/\hbar =0\right)$, with the |1〉↔|2〉 transition frequency slightly offset from resonance. The non-zero detuning Δ is set to $1.5{\;\mathrm{ps}}^{-1}$ and the two-photon resonance condition indicates a biexciton energy shift $E_{B}/\hbar$ value of $-3{\;\mathrm{ps}}^{-1}$. The free-space pump field Rabi frequency $\Omega_{0a}$ is fixed at $0.9{\;\mathrm{ps}}^{-1}$, the same value used in Figure 6. According to the dressed-state analysis, under the two-photon resonance condition with a positive detuning Δ, the spectra exhibit a central resonance, along with three doublets of sideband resonances. These features appear consistently across all configurations. The analysis predicts, and the spectra confirm, that these resonances arise at frequency mismatch values $\delta =\pm \sqrt{\Delta^{2}+8{\left|\Omega_{eff}\right|}^{2}}$, $\pm \left(\Delta +\sqrt{\Delta^{2}+8{\left|\Omega_{eff}\right|}^{2}}\right)/2$, $\pm \left(\Delta -\sqrt{\Delta^{2}+8{\left|\Omega_{eff}\right|}^{2}}\right)/2$ and 0, as shown in the left diagram of Figure 2a. Thus, the spacing between the resonances of the outer doublets, on either side of the spectrum, is half the separation between the central pair of resonances. Notably, the sideband resonances correspond to the positions of the three minima and three maxima detected on the Re[χSQD(1)] spectrum. As in the previous case, at large interparticle distances, all spectra exhibit antisymmetric lineshapes that are nearly identical across different configurations. However, at shorter distances, where interactions between system components become more pronounced, this symmetry is broken, leading to configuration-dependent spectral features. Moreover, a decrease in interparticle distance increases the spacing between resonances, in agreement with Figure 5, which shows that $\left|\Omega_{eff}\right|$ decreases monotonically. Compared to the combined one-photon and two-photon resonance case, these resonances show lower, yet more distance-sensitive, magnitude values. Across configurations, the resonance spread along the frequency axis is widest in configuration (b), narrower in (a), and minimal in (c), where all resonances nearly overlap, indicating negligible variation. This behavior is mainly attributed to differences in the $\left|\Omega_{eff}\right|$ parameter, particularly the contribution related to the MNP volume. More specifically, configurations (a) and (b) share the same MNP volume, but configuration (b) exhibits a stronger interaction, reflected in a higher $\left|\Omega_{eff}\right|$ value and broader spectra, as seen in Figure 5.

**Figure 9:**
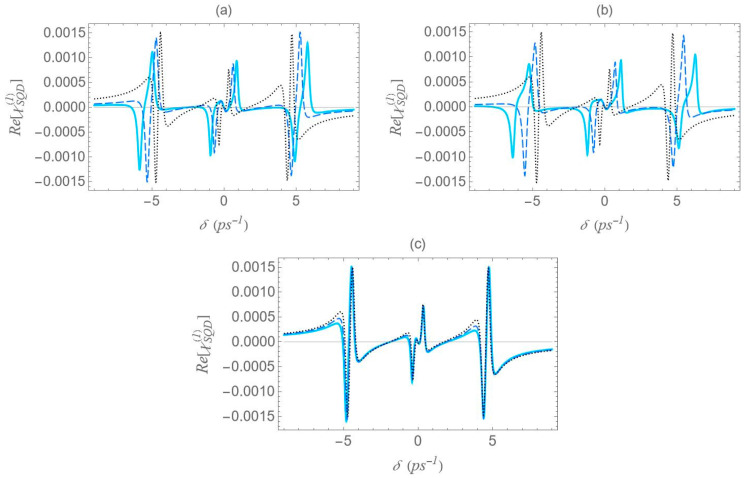
Dispersion spectrum of the SQD, Re[χSQD(1)], under the two-photon resonance condition $\left(2\Delta +E_{B}/\hbar =0\right)$, for Rabi frequency $\Omega_{0a}=0.9{\;\mathrm{ps}}^{-1}$, pump field detuning $\Delta =4{\;\mathrm{ps}}^{-1}$, and biexciton energy shift $E_{B}/\hbar =-8{\;\mathrm{ps}}^{-1}$.

In Figure 9, the Re[χSQD(1)] spectrum is presented under the two-photon resonance condition $\left(2\Delta +E_{B}/\hbar =0\right)$, using the same physical parameters as in Figure 8, but with a higher pump field detuning $\left(\Delta =4{\;\mathrm{ps}}^{-1}\right)$. Under this condition, the corresponding biexciton energy shift is $E_{B}/\hbar =-8{\;\mathrm{ps}}^{-1}$. The same analytical expressions used in the analysis of Figure 8 accurately predict the positions of the seven resonances: one central peak and three sideband doublets. All general observations from Figure 8 remain valid. However, increasing the detuning parameter Δ from 1.5 to $4{\;\mathrm{ps}}^{-1}$, for a fixed interparticle distance, leads to the following notable effects across all spectra: First, the amplitude of the inner sideband resonances decreases, while that of the outer resonances increases, showing an opposite trend. Second, the span of each outer doublet narrows, and their distance from the adjacent inner resonances becomes smaller. These effects are consistent with the analytical expressions for the resonance positions discussed earlier and highlight the impact of pump detuning on the spectral structure. As supported by the preceding analysis, an increase in the exciton detuning Δ significantly reduces the sensitivity of the spectral lineshape to the geometrical characteristics of the MNP.

**Figure 10:**
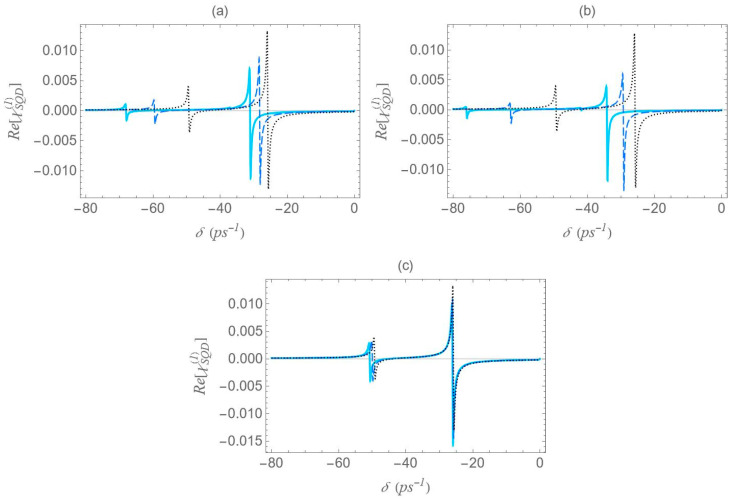
Dispersion spectrum of the SQD, Re[χSQD(1)], under the one-photon resonance condition (Δ=0), in the case with Rabi frequency $\Omega_{0a}=12.4{\;\mathrm{ps}}^{-1}$, and biexciton energy shift $E_{B}/\hbar =-30{\;\mathrm{ps}}^{-1}$. All other parameters are the same as in Figure 6.

In Figure 10, we present the dispersion spectra at various interparticle distances for the different configurations, under the one-photon resonance condition (Δ=0), but away from the two-photon resonance condition $\left(2\Delta +E_{B}/\hbar \neq 0\right)$. Here, a significantly higher free-space pump field Rabi frequency $\left(\Omega_{0a}=12.4{\;\mathrm{ps}}^{-1}\right)$ and a biexciton energy shift $E_{B}/\hbar =-30{\;\mathrm{ps}}^{-1}$ are considered. In contrast to Figure 6, Figure 8 and Figure 9, the spectra here exhibit no symmetry. For each interparticle distance, only two dominant, dispersion-like resonances are observed, both located at negative δ values. The separation between these resonances can be roughly approximated by $2\left|\Omega_{eff}\right|$. Importantly, both the spectral position and amplitude of the resonances exhibit a much stronger dependence on interparticle distance compared to the previously examined cases. More precisely, a decrease in interparticle distance results in a noticeable increase in the separation between the resonances, accompanied by a significant reduction in their amplitude. This behavior marks a clear departure from the predictions of the dressed-state model. This deviation from the dressed-state picture can be attributed to the model’s underlying assumption that the detuning is much smaller than the Rabi frequency, a condition necessary for strong field–matter coupling and efficient dressed-state mixing. However, in the current regime, where the detuning is large compared to the Rabi frequency (in our case, we have a significantly off-resonant $|1\rangle ↔|2\rangle$ transition), the system-field interaction becomes weak, and state mixing is insufficient for the dressed-state picture to describe the system’s dynamics. Instead, a different approach, such as adiabatic or perturbative approximations, where the influence of the field is treated as a weak perturbation, is more suitable for this regime. Comparing the spectra across configurations, we reach similar conclusions to those in Figure 6, Figure 8 and Figure 9: configuration (b) shows the widest spread of resonance positions across different interparticle distances, configuration (a) displays a moderate spread, and in configuration (c) all resonances appear to nearly overlap with each other.

These results show that the geometry and interparticle distance strongly influence the dispersion response of the system. The outward shift and broadening of the resonances with decreasing R directly reflect the enhancement of $\left|\Omega_{eff}\right|$ and the increase in the local-field intensity arising from the plasmon–exciton coupling. This behavior confirms the key role of hybridization strength in shaping the optical response and governing the spectral tunability of the system.

We now focus on the spectra of the imaginary part of the optical susceptibility of the SQD, Im[χSQD(1)], which governs the absorption and gain spectra of the probe field as a function of the pump–probe frequency mismatch δ. In Figure 7, under one-photon and two-photon resonance conditions $\left(\Delta =E_{B}=0\right)$, for pump field Rabi frequency $\Omega_{0a}=0.9{\;\mathrm{ps}}^{-1}$, the spectra exhibit three resonances, one central and a doublet of sideband resonances, consistent with the behavior observed in the spectra of Re[χSQD(1)] (Figure 6), as predicted by the dressed-state model. As addressed in the left diagram of Figure 2b, the dressed-state picture for this limiting case predicts the presence of three resonances at δ=0, $\pm \sqrt{2}\left|\Omega_{eff}\right|$. More specifically, while for pump–probe mismatch $\delta =\;\pm \sqrt{2}\left|\Omega_{eff}\right|$, the spectrum of Re[χSQD(1)] manifests its minimum and maximum values and the exciton–biexciton system displays zero probe absorption (Im[χSQD(1)]=0). For large interparticle distances, all spectra display highly symmetric profiles, but this symmetry is broken as the distance decreases. Similarly to Figure 6, as the interparticle distance decreases, the separation between the sideband resonances $\left(\approx 2\sqrt{2}\left|\Omega_{eff}\right|\right)$ increases, enhancing the width of the gain regions. Higher absorption occurs at positive pump–probe frequency mismatch and shorter interparticle distances, while substantially enhanced gain is achieved for negative values of δ. Examining the different configurations, we deduce that broader gain regions are achieved in configuration (b) compared to configuration (a), while in configuration (c), all resonances coincide and the gain region is substantially shrunk. The maximum gain and absorption coefficients are obtained in configuration (b), where the stronger effective coupling enhances the nonlinear optical response.

**Figure 11:**
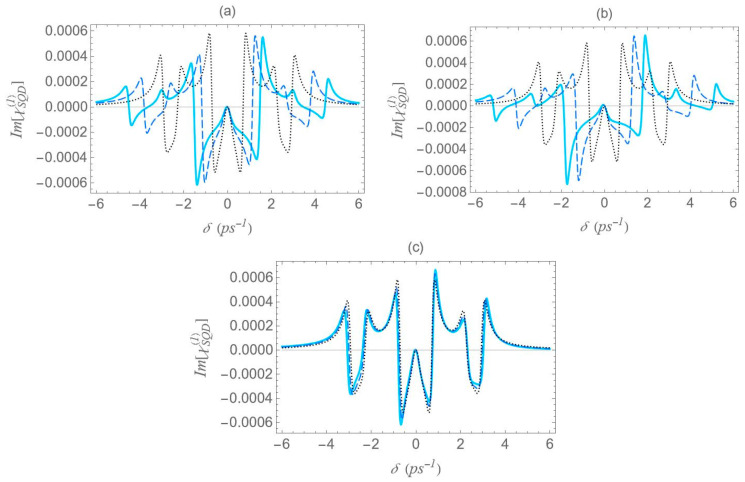
Absorption spectrum of the SQD, Im[χSQD(1)], under the two-photon resonance condition $\left(2\Delta +E_{B}/\hbar =0\right)$, for Rabi frequency $\Omega_{0a}=0.9{\;\mathrm{ps}}^{-1}$, pump field detuning $\Delta =1.5{\;\mathrm{ps}}^{-1}$, and biexciton energy shift $E_{B}/\hbar =-3{\;\mathrm{ps}}^{-1}$.

In Figure 11, we present the gain spectra for configurations (a), (b), and (c) under the two-photon resonance condition $\left(2\Delta +E_{B}/\hbar =0\right)$, but with an off-resonant |1〉↔|2〉 exciton transition. As in Figure 8, the non-zero detuning Δ is set to $1.5{\;\mathrm{ps}}^{-1}$ and the two-photon resonance condition indicates a biexciton energy shift $E_{B}/\hbar$ value of $-3{\;\mathrm{ps}}^{-1}$. Consistent with Figure 2, all spectra validate the dressed-state picture, with the exciton–biexciton system manifesting zero absorption at the predicted δ values. Under these resonance conditions ($2\Delta +E_{B}/\hbar =0$, Δ≠0), we observe three distinct gain regions centered approximately at δ=0, $\pm \left(\Delta +3\sqrt{\Delta^{2}+8{\left|\Omega_{eff}\right|}^{2}}\right)/4$. The central gain region spans a width of approximately $\sqrt{\Delta^{2}+8{\left|\Omega_{eff}\right|}^{2}}-\Delta$, whereas each sideband gain region spans $\left(\sqrt{\Delta^{2}+8{\left|\Omega_{eff}\right|}^{2}}-\Delta \right)/2$, half the width of the central region. As the interparticle distance decreases, the range of the gain region increases, due to the enhancement of the $\left|\Omega_{eff}\right|$ parameter, as seen in Figure 5, with the (b) configuration yielding the broader gain region. Once again, in the regime of large interparticle separations, the spectra exhibit highly symmetric profiles; however, as the distance decreases, this symmetry gradually breaks, resulting in a higher gain coefficient for negative δ values and a higher absorption coefficient for positive δ values. Compared to the case where both transitions are characterized by zero detuning, these resonances demonstrate lower overall magnitudes, but their lineshape presents a more pronounced dependence on distance.

**Figure 12:**
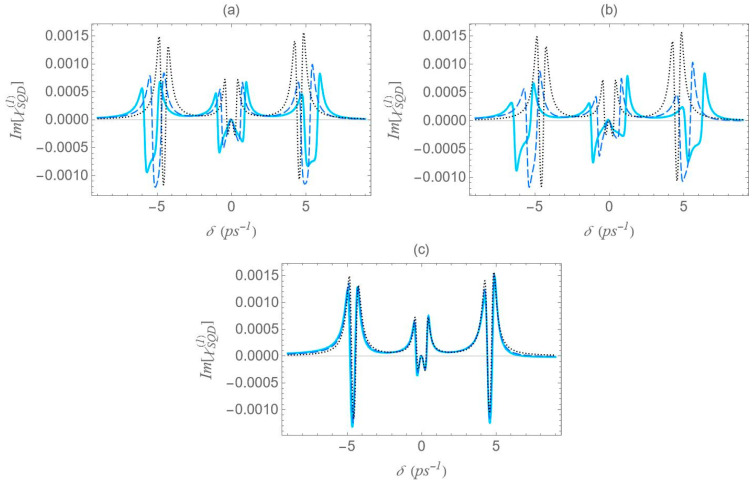
Absorption spectrum of the SQD, Im[χSQD(1)], under the two-photon resonance condition $\left(2\Delta +E_{B}/\hbar =0\right)$, for Rabi frequency $\Omega_{0a}=0.9{\;\mathrm{ps}}^{-1}$, pump field detuning $\Delta =4{\;\mathrm{ps}}^{-1}$, and biexciton energy shift $E_{B}/\hbar =-8{\;\mathrm{ps}}^{-1}$.

In Figure 12, we present the absorption/gain spectra for configurations (a), (b), and (c) under the two-photon resonance condition $\left(2\Delta +E_{B}/\hbar =0\right)$, using the same physical parameters as in Figure 6, but with a higher pump field detuning $\left(\Delta =4{\;\mathrm{ps}}^{-1}\right)$. All observations made for Figure 11 remain valid, including the analytical expressions used to estimate the positions and widths of the gain regions. However, the increase in the detuning parameter Δ from 1.5 to $4{\;\mathrm{ps}}^{-1}$, for a fixed interparticle distance, significantly enhances the spectral minima and maxima. It also increases the separation of the outer sideband resonances from the spectral center, while simultaneously narrowing the gain regions around the sideband resonances.

**Figure 13:**
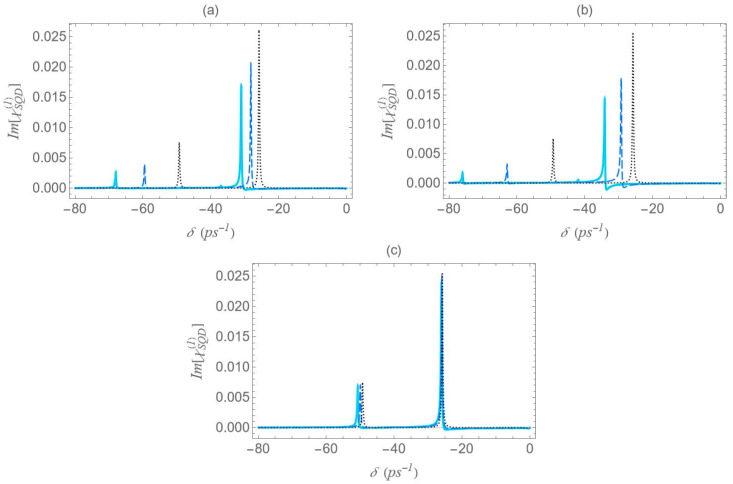
Absorption spectrum of the SQD, Im[χSQD(1)], under the one-photon resonance condition (Δ=0), in the case with Rabi frequency $\Omega_{0a}=12.4{\;\mathrm{ps}}^{-1}$, and biexciton energy shift $E_{B}/\hbar =-30{\;\mathrm{ps}}^{-1}$. All other parameters are the same as in Figure 6.

Lastly, Figure 13 shows the absorption/gain spectra calculated with the same parameters as in the dispersion spectra of Figure 10. Similarly to Figure 10, the spectra exhibit no apparent symmetry. The spectral lineshape features two dominant Lorentzian-shaped resonances, located at the same spectral positions as those in Figure 4. The distance between them can be roughly approximated by $2\left|\Omega_{eff}\right|$ and this is why the higher separation is observed for configuration (b), at the center-to-center distance R=12.5 nm. Consistent with the findings of Figure 4, the resonance amplitudes show a considerably stronger dependence on the interparticle distance compared to those in Figure 7, Figure 11 and Figure 12, with a decrease in interparticle distance resulting in a pronounced reduction in absorption amplitude. At large interparticle distances, no gain is observed. At shorter separations, however, appreciable gain appears for configuration (b) of Figure 1, the prolate nanospheroid with its polar axis perpendicular to the structure’s symmetry axis, at the lowest interparticle distance examined R=12.5 nm. This configuration also produces the broadest gain band of all cases studied. Meanwhile, the absorption bands’ amplitudes are drastically diminished.

In these spectra, the emergence of gain peaks and spectral asymmetries arise from the exciton–plasmon coupling mechanism. As the MNP aspect ratio or the interparticle distance varies, the hybridization modifies the energy exchange between absorption and stimulated emission, leading to enhanced optical gain and distinct spectral reshaping. These effects are clear signatures of strong exciton–plasmon interaction in the hybrid system.

**Figure 14:**
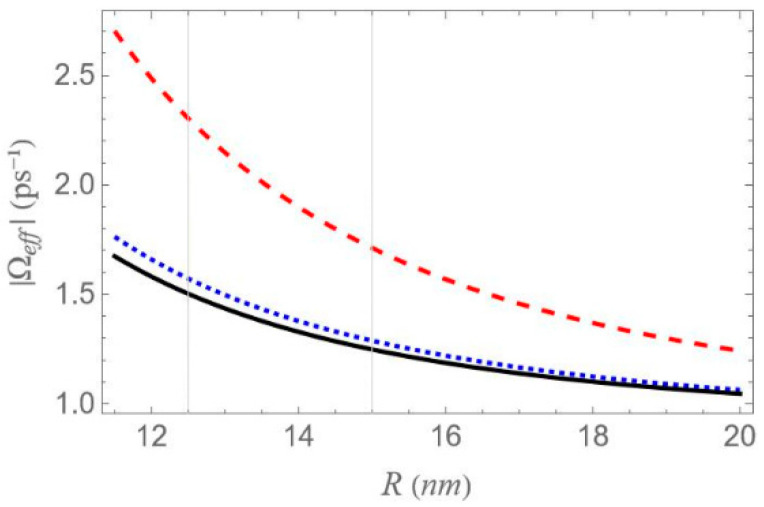
The absolute value of the effective Rabi frequency, $\left|\Omega_{eff}\right|$, as a function of the interparticle distance, R, for Ag MNPs. The dashed red, dotted blue, and solid black curves correspond to configurations (a), (b), and (c) of Figure 1, respectively, assuming a pump field Rabi frequency $\Omega_{a}=0.9{\;\mathrm{ps}}^{-1}$. Compared with Au, Ag exhibits a 35% enhancement of $\left|\Omega_{eff}\right|$ at short distances, due to its larger negative permittivity and lower damping, leading to stronger exciton–plasmon coupling. All other parameters are identical to those used in the Au-based calculations of Figure 5 in the main text.

**Figure 15:**
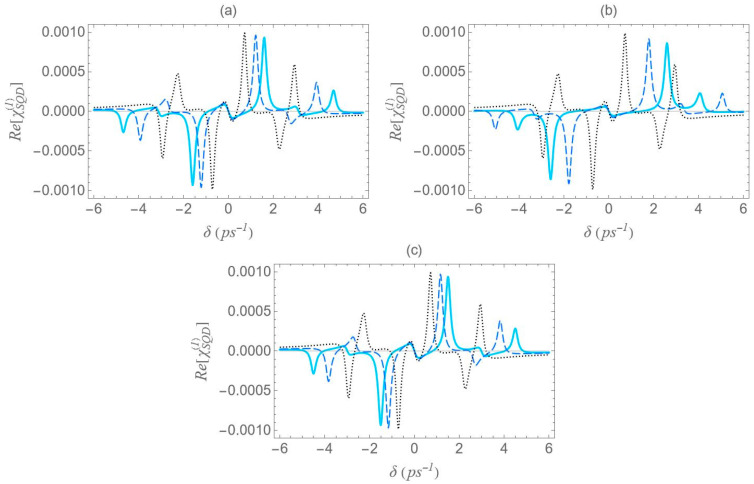
Dispersion spectrum of the SQD, Re[χSQD(1)], under the two-photon resonance condition $\left(2\Delta +E_{B}/\hbar =0\right)$, for Ag MNPs, with pump field Rabi frequency $\Omega_{0a}=0.9{\;\mathrm{ps}}^{-1}$, detuning $\Delta =1.5{\;\mathrm{ps}}^{-1}$, and biexciton energy shift $E_{B}/\hbar =-3{\;\mathrm{ps}}^{-1}$. The overall resonant structure is the same as in Au case (Figure 8), but the effective coupling is stronger and less geometry-dependent. For configurations (a,c), the spectra nearly overlap, confirming the weak geometry dependence in the Ag case. In configuration (b), the secondary sideband peaks move slightly toward the spectral center at short distances, consistent with phase-sensitive plasmon–exciton interference in the low-loss Ag regime. All other parameters are identical to those in Figure 8.

Gold nanoparticles were chosen for the main analysis because their plasmonic response offers a broader and more controllable tunability of the exciton–plasmon interaction across the parameter space examined. To complement these results, the corresponding calculations for Ag nanoparticles are provided in Figure 14 and Figure 15, obtained under identical excitation conditions. Owing to its more negative permittivity and lower losses, Ag produces a noticeably stronger local-field feedback, leading to an ∼35% enhancement of the effective Rabi frequency $\left|\Omega_{eff}\right|$ at short separations (Figure 14). Despite this quantitative increase, the qualitative dependence of $\left|\Omega_{eff}\right|$ on the interparticle distance remains consistent with the Au case.

The dispersion spectra shown in Figure 15 further confirm that the characteristic spectral structure persists when Ag is used instead of Au. Minor configuration-dependent differences arise due to the low-loss nature of Ag: in configuration (b) the secondary sidebands shift slightly toward the spectral center at smaller values of R, whereas in configurations (a) and (c) the spectra nearly coincide. These features reflect the stronger plasmon–exciton interference induced by the Ag nanoparticle. Overall, the Ag results of Figure 14 and Figure 15 support the same physical conclusions drawn from the Au analysis while illustrating the expected quantitative strengthening of the coupling for Ag.

## 5. Conclusions

We have presented a theoretical study of the probe absorption/gain and dispersion in a strongly driven, near-resonant exciton–biexciton SQD–gold spheroidal MNP hybrid nanostructure. Using the density matrix formalism, we examined how the optical spectra vary with MNP geometry, interparticle separation, pump-field detuning, and biexciton energy shift, under regimes that include either one-photon or two-photon resonance, or both simultaneously. The spectral characteristics are explained using the dressed-state picture, providing deeper insight into the optical behavior of the strongly pumped three-level cascade system coupled to the MNP.

In the one-photon regime, but away from two-photon resonance, the obtained spectra feature two dominant resonances at negative frequency mismatch values. Under two-photon resonance, the dispersion (absorption/gain) spectra exhibit antisymmetric (symmetric) lineshapes at sufficiently large interparticle distances. However, this symmetry gradually breaks as the dipole–dipole interaction between the two components of the hybrid nanostructure increases. The interaction between the SQD and the MNP is markedly enhanced for low center-to-center distances, as well as when the MNP has a large volume and a pronounced shape anisotropy, with its long semiaxis oriented in parallel with the field polarization direction. This enhanced coupling leads to an outward shift of the sideband resonances. At large interparticle separations, no optical gain is observed; however, at smaller distances, considerably enhanced gain is typically manifested in the presence of a prolate MNP whose polar axis is aligned with the interparticle axis. This configuration supports the broadest gain bandwidth among all cases investigated. Our findings illustrate how the interplay between resonant driving conditions, quantum-level structure, and nanoparticle geometry enables the tunability of the optical response of SQD-MNP hybrid systems, with potential applications in nanoscale optical devices, including amplifiers, switches, and sensors.

The present framework may be further extended to examine other nonlinear responses, such as FWM and self-Kerr nonlinearities, in the same hybrid geometry. It can also be adapted to other plasmonic nanostructures, such as nanostars and nanoprisms, whose anisotropic field distributions may enable even stronger and more tunable exciton–plasmon coupling. For SQDs, one may use asymmetric quantum structures, which also allow second-order nonlinear optical effects, such as nonlinear optical rectification, second-harmonic generation and sum- or difference-frequency generation. Such directions offer promising opportunities for designing advanced nanoscale photonic elements based on geometry-controlled hybrid interactions.

## Data Availability

The raw data supporting the conclusions of this article will be made available by the authors on request.
